# Culture and its impact on research

**DOI:** 10.1038/s44319-025-00453-1

**Published:** 2025-04-22

**Authors:** Frank Gannon

**Affiliations:** QIMR Berghofer Medical Research Institute, Brisbane, QLD Australia

**Keywords:** Economics, Law & Politics, History & Philosophy of Science, Science Policy & Publishing

## Abstract

The career structure for researchers is different to that of clinicians. This results in a culture that is more focused on individual success and impedes rapid implementation.

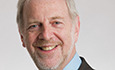

I was at a cancer meeting in Brisbane when the question about the culture of a community occurred to me. The meeting was well attended with researchers and clinicians and had sessions that included both basic research and clinical research projects. During a session on breast cancer, one speaker, a clinician, gave extensive information about and credit to the work of other research teams and ongoing clinical trials around the world before discussing their own treatment variation in an investigator-led clinical trial. It was also obvious from the presentation that their main concern was their patients and how to achieve a better outcome for their patients.

The talks in the same session by life scientists were more familiar in style and content to those expected at any standard scientific meeting. The speakers said that the area they were addressing was shown to be important, the target gene was “theirs” owing to key findings in their earlier papers, and the preclinical work in their lab—mostly cell culture—showed great promise for developing new therapies. The latter point was reinforced by the fact that the work had been submitted to a high-impact journal. In contrast with the clinician’s presentation, there was no reference to alternative targets for the selected disease and how studies by other groups were progressing, perhaps even in the clinic. Nor was the work of others on “their gene” cited in a clear manner other than by the usual “it has been shown” phrase. If there had been earlier unsuccessful attempts by the presenter to show an effect on the target pathway, they were not shared with the audience.

The researchers’ message was: “I am going to solve this problem.” The clinician’s message was: “We are working together to solve the problem.” I think the reason for their different attitude is different research cultures. A scientist is working in a very competitive and insecure environment. A clinician has a reasonably secure job with a clear career path where each step up brings more stability and increasingly better-paid contracts. The clinicians’ success and long-term reputation depend on how well their patients do while the scientist needs a long publication list in order to advance on the career ladder.

Scientists start their training with a long precarious phase of learning by doing, having luck—or not—with their supervisor and their research topic and their career pathway is not signposted as is the case for a physician who eventually gets formal professional approval. Scientist will get a job or a promotion mostly based on grants, which, in turn, largely depends on their publications. They monetize—that is, get contract renewals—their work through papers which have to emphasize the importance of their individual contribution and of their laboratory. They have to “sell” their research, for example, by presentations at meetings. They are pushed into a mode of behavior that makes generosity difficult. This has been termed the “Gollum effect”, characterized by “inappropriate possessiveness over research topics and resources” (Gould and Valdez, 2022).

The cultural consequences of competition inevitably trickle down to the laboratory. The fear of being scooped increases as the research team comes closer to having enough results they need for a good publication. It pushes groups to go early and publish incomplete stories. An analysis of structural biology publications using the Protein Data Base showed that scientists concerned about being scooped tended to rush to publish papers in which the research had not been finalized (Hill and Stein, 2025). Follow-up papers will have a lower impact but the aim of claiming ‘to be the first’ remains protected. So-called “salami” publications, where a research project is parceled into as many individual papers as possible, is another consequence of this competitive attitude.

The life-science community has two drivers that result in this individualistic culture. “Publish or Perish” is one. Without publications, careers fail and alternative work options have to be explored. The publications must have clear ownership either by first or last authorship and sometimes sharp elbows are employed to achieve this. Equal first—or last—authorship would make the decision of the laboratory head easier, but I have witnessed bitter arguments over the name order even with an asterix that shows equal input. The pressure to publish rather than perish creates a laboratory culture that does not encourage collaboration. The politics of collaboration with external groups with similar skills is even more fraught.

The second driver is “Publish and Prosper”. This may seem like the consequence of the first one, but it is more than that, even for well-established researchers. Prospering in this context means real money, income from a patent, invitations to become a consultant for biotech or pharma companies, opportunities to speak at meetings, and of course promotions or job offers. All these outcomes require a clear linkage between the research that is published and an individual claiming credit for it. Again, this drives behavior that can place individual success ahead of collaboration or generosity in recognizing the work of others. The ultimate goal of having an impact that benefits patients becomes secondary or just forgotten.

At a time when funding systems emphasize translation, it is unfortunate that the research culture in the life sciences is primarily inward looking rather than focused on getting the best outcome for society as quickly as possible. Correcting this flaw would have to come from selection committees probing the applicant for proof of collaboration within and outside the laboratory. In addition, more engagement with patients or user groups should also have a bearing on funding decisions so as to change the inward-looking culture. The Covid period showed that openness and collaboration can accelerate progress and bring direct benefit to the society that supports the research. This should apply to all areas if the results and insights of clever researchers are to be harnessed. The next time I’m sitting at a research conference, I will be more tolerant of a presentation that provides a lot of background information and credit to others before it moves on to the presenter’s own results. In the past, I would have judged this as a cover for inadequate new data. Now it may well be a hallmark of an open and generous researcher who is more interested in moving his research into applications.

## Supplementary information

Peer Review File
