# Supplementary material for: Culture and its impact on research
Source: EMBO Rep. 2025 Apr 22;26(10):2487–8. doi: 10.1038/s44319-025-00453-1 (PMC12117146; doi:10.1038/s44319-025-00453-1)
Supplement: Supplementary file 1 — Peer Review File [file 44319_2025_453_MOESM1_ESM.pdf]

EMBO reports

The culture and impact of research

Frank Gannon

Corresponding author(s): Frank Gannon (FGannon@qimrberghofer.edu.a, proinnsiasg@hotmail.comu)

Review Timeline:

Submission Date:	7th Apr 25
Accepted:	8th Apr 25

Editor: Holger Breithaupt

Transaction Report: This article type is not peer reviewed.
